# Impaired muscarinic modulation of the rat prelimbic cortex in neuropathic pain is sexually dimorphic and associated with cold allodynia

**DOI:** 10.3389/fncel.2023.984287

**Published:** 2023-02-09

**Authors:** Taylor Jefferson, Haram R. Kim, Marco Martina

**Affiliations:** Department of Neuroscience, Feinberg School of Medicine, Northwestern University, Chicago, IL, United States

**Keywords:** chronic pain, mPFC, medial PFC, acetylcholine, pyramidal cell, SNI (spared nerve injury)

## Abstract

Cholinergic modulation of the brain cortex is critical for cognitive processes, and altered cholinergic modulation of the prefrontal cortex is emerging as an important mechanism of neuropathic pain. Sex differences in pain prevalence and perception are well known, yet the precise nature of the mechanisms responsible for sexual dimorphism in chronic neuropathic pain are poorly understood. Here we investigated potential sex differences in cholinergic modulation of layer five commissural pyramidal neurons of the rat prelimbic cortex in control conditions and in the SNI model of neuropathic pain. We discovered that cholinergic modulation is stronger in cells from male compared with female rats, and that in neuropathic pain rats, cholinergic excitation of pyramidal neurons was more severely impaired in males than in females. Finally, we found that selective pharmacological blockade of the muscarinic M1 subunit in the prefrontal cortex induces cold sensitivity (but not mechanical allodynia) in naïve animals of both sexes.

## Introduction

Despite recent progress, the cellular mechanisms of chronic pain are still poorly understood, which prevents the development of efficacious therapies. Several factors have contributed to the slow therapeutic progress, and two in particular are likely to have major roles. One factor is that the sexual dimorphism of chronic pain and of the underlying cellular mechanisms (Mogil, 2020) have been overlooked. Indeed, chronic pain prevalence is higher in females (Pitcher et al., 2019) yet most experimental work on animal models has been performed on males; while this approach was designed to avoid the potential complications caused by the physiological changes during the estrous cycle, it likely contributed to failures in clinical trials because the efficacy of treatments may differ largely in females and males (Reckziegel et al., 2021). The other factor is an often too narrow focus on the peripheral nervous system and the spinal cord. While acute pain is the result of hyperactivity of nociceptors, work over the past ∼20 years has shown that supraspinal mechanisms are central in the development of chronic pain (Apkarian et al., 2009). Establishing the involvement of a brain structure in the pathogenesis of pain is made more complex by the fact that pain is a multidimensional experience with sensory, motivational, and affective components; consequently, multiple brain areas are necessary, but not sufficient, for pain perception. Among the brain areas that are involved in pain perception and chronification, the medial prefrontal cortex (mPFC) has recently attracted much attention because it is involved in all the different aspects of the chronic pain phenotype. Numerous papers have identified deactivation of mPFC as a causal contributor to both the sensory and cognitive components of the pain phenotype (Sun and Neugebauer, 2011; Lee et al., 2015; Zhang et al., 2015). Multiple factors contribute to the pain-associated mPFC deactivation, including increased GABAergic inhibition (Ji et al., 2010; Zhang et al., 2015), decreased excitatory drive (Kelly et al., 2016; Kelly and Martina, 2018) and decreased intrinsic excitability (Wang et al., 2015). mPFC modification in neuropathic pain is not purely functional but also includes morphological changes (Metz et al., 2009; Cordeiro Matos et al., 2018; Shiers et al., 2018). Intriguingly, some cortical rearrangement associated with neuropathic pain exhibit sexual dimorphism. For example, the length of axon initial segments of L5 pyramidal neurons of the infralimbic cortex is reduced in male, but not female mice, and cognitive impairments are stronger in males than in females (Shiers et al., 2018). Additionally, in neuropathic pain conditions, excitability of prelimbic cortex layer five parvalbumin-positive interneurons is increased in male, but not female, mice (Jones and Sheets, 2020). Several papers have reported sexual dimorphism in cholinergic modulation of memory functions; for example, muscarinic blockade of the ventral hippocampus caused memory impairments in female rats but had no significant effect in males (Hall et al., 2017). Additionally, spontaneous acetylcholine release in the mPFC during a 24 h cycle is stronger in female compared to male rats (Takase et al., 2009) and very recent data show that in male transgenic animals with reduced expression of the acetylcholine vesicular transporter, are impaired in both short- and long-term memory tasks, but females only show impaired long-term memory (Capettini et al., 2011). We previously showed that excitatory M1-mediated cholinergic modulation is severely impaired in presumed commissural pyramidal neurons of male neuropathic pain rats 1 week after peripheral injury (Radzicki et al., 2017), which may play an important role in the overall cortical deactivation. Here we show that cholinergic modulation of the rat prelimbic cortex (PLC) in neuropathic pain conditions is differentially affected in male and female rats and that selective M1-mediated inhibition of the mPFC has a causal role in pain perception in both males and females.

## Materials and methods

### Animals

All animal procedures were performed with approval of the Northwestern University Institutional Animal Care and Use Committee. Male and female Long-Evans rats (Charles River, Wilmington, MA, USA) were housed in conventional rodent housing with unlimited access to food and water and kept on a 12/12 h light/dark cycle. All behavioral testing was performed during the light cycle.

### Neuropathic pain animal model

Spared Nerve Injury (SNI) (Decosterd and Woolf, 2000) is a widely used model of neuropathic pain. The main advantages of this model are its robustness, as all animals develop neuropathic pain, and the fact that it produces in rodents both sensory and emotional/cognitive symptoms of neuropathic pain. Briefly, rats (35-day old) were anesthetized using gas anesthesia (isoflurane 2–3% in O_2_). The left sciatic nerve was exposed at the level of the trifurcation of the peroneal, tibial, and sural branches. The peroneal and tibial nerves were ligated and a 1–2 mm-long segment at the proximal end was removed, while the sural nerve was untouched. For sham surgery, the nerves were exposed but left untouched.

### Stereotaxic surgery and virus infusion

A total of 22–24 day-old Long Evans rats of both sexes, were anesthetized with isoflurane inhalation (4%) and secured in a stereotaxic frame (David Kopf Instruments, Tujunga, CA, USA). Isoflurane concentration was reduced to 2% with a flow rate of 0.8 L/min. Hair was removed with a hair removal cream and the skin was cleaned with alcohol and a 10% povidone-iodine solution. Eye ointment (Covetrus, Portland, ME, USA) was applied to the eyes to prevent dryness. Under magnification, a midline incision was made over the dorsal surface of the skull. Injections were performed using a (Hamilton, Reno, NV, USA) syringe (10 μl). Coordinates to target the left hemisphere, prelimbic cortex were +2.5 mm anterior to bregma; −0.5 mm lateral to midline; 3.5 mm ventral to the surface of bregma. A volume of 450 nl of retrograde virus expressing a fluorescent tag (pAAV-hsyn-EGFP, 450 nl, titer: 1.7 × 10^13^ GC/ml; Addgene, Watertown, MA, USA, Plasmid #50465) was delivered unilaterally at a rate of 150 nl/min. The syringe was left in place for a minimum of 5 min after each injection. After 5 min, the needle was retracted 100 μm and remained in place for an additional 3 min. Once the needle was fully retracted, the skin was sutured with sterile monofilament suture (4–0). A total of 4% Lidocaine cream was applied to the skin post-operatively. Following at least 21 days recovery period, rats were sacrificed for *ex vivo* electrophysiological recordings or *in situ* hybridization.

### Electrophysiological recordings

#### Slice preparation

Rats were anesthetized with 4% isoflurane and sacrificed by decapitation 7 days after the Sham/SNI surgery (40–45 day-old at the time of sacrifice). The brain was removed from the skull and 300-μm thick coronal slices of the medial prefrontal cortex were cut in ice-cold, low calcium, high magnesium artificial cerebral spinal fluid (aCSF) containing (in mM): 125 NaCl, 25 glucose, 25 NaHCO_3_, 2.5 KCl, 1.25 NaH_2_PO_4_, 0.5 CaCl_2_ and 7 MgCl_2_, equilibrated with 95% O_2_, and 5% CO_2_ (pH 7.4). Slices were then stored for ∼20 min at 35°C and then allowed to recover at room temperature (22–24°C) for at least 30 min in the same solution.

#### Whole-cell patch clamp recording

Slices were visualized using a (Zeiss, Dublin, CA, USA) upright microscope using with oblique infrared illumination and a water immersion 40X objective connected to a video camera (Dage-MTI, Michigan City, IN, USA). Commissural L5 pyramidal cells were visually identified according to location and expression of EGFP. Patch clamp recordings were performed with an Axopatch-200B amplifier controlled by pClamp 9.0 software using a Digidata 1322A digitizer (Axon Instruments, Union City, CA, USA). Signals were filtered at 10 kHz and digitized at 20 kHz. Pipettes were pulled from thick wall borosilicate glass (1.5 mm outer diameter; Sutter Instruments, Novato, CA, USA) using a horizontal puller (Sutter Instruments P-97) and were filled with a solution composed of (in mM): 140 K-gluconate, 8 NaCl, 2 MgCl_2_, 0.1 EGTA, 10 HEPES, 2 Mg-ATP, and 0.2 Na_3_-GTP (pH 7.3 with KOH). The extracellular bath solution (aCSF) contained (in mM): 125 NaCl, 25 glucose, 25 NaHCO_3_, 2.5 KCl, 1.25 NaH_2_PO_4_, 2 CaCl_2_ and 1 MgCl_2_ and with 2.5 mM kynurenic acid, 0.1 picrotoxin and equilibrated with 95% O_2_ and 5% CO_2_. Recordings were performed at 28–31°C (TC-324B control unit, Warner Instruments, Holliston, MA, USA) in the presence of 2.5 mM kynurenic acid and 0.1 mM picrotoxin to block fast glutamatergic and GABAergic synaptic currents. Pipette resistances in working solutions ranged from 3 to 5 MΩ yielding series resistances for whole cell recordings of 10–15 MΩ. Series resistance was not compensated. Resting membrane potentials were measured immediately after obtaining electrical access; only neurons with resting membrane potential negative to −60 mV were accepted for the study. Firing properties were determined using depolarizing current injections (0–500 pA, 25 pA steps, 2 s duration; 0.3 Hz) from a holding potential of −70 mV. Acetylcholine (ACh) was applied using a home-made, gravity driven, quartz-pipe delivery system, positioned ∼500 μm above the slice. ACh (1 mM final concentration) was dissolved in a HEPES buffered extracellular solution (in mM: 138 NaCl, 2.5 KCl, 1 MgCl_2_, 2 CaCl_2_, 25 glucose, 10 HEPES, pH 7.3 with NaOH) containing 2.5 mM kynurenic acid and 0.1 mM picrotoxin.

#### Drug stock solutions

Picrotoxin (100 mM) was prepared in dimethylsulfoxide (DMSO) and stored at −20°C. Kynurenic acid (500 mM) was prepared in 1 N NaOH and stored at 4°C. Pirenzepine dihydrochloride (10 mM), was prepared in water and stored at −20°C. Acetylcholine (100 mM) was prepared in water and stored at 4°C. Working solutions were prepared freshly on each experimental day. All chemicals were from (Sigma-Aldrich, St. Louis, MO, USA), except pirenzepine (Tocris, Bristol, United Kingdom).

### *In situ* hybridization and immunostaining

#### Section preparation

Rats were deeply anesthetized with pentobarbital sodium solution (150 mg/kg; ip) and transcardially perfused with ∼100 ml of 0.9% saline followed by ∼500 ml phosphate buffered 4% paraformaldehyde. The brains were post-fixed in 4% paraformaldehyde for 1 h, and then equilibrated to 30% sucrose in phosphate buffer saline (PBS) containing 0.1% diethyl pyrocarbonate (DEPC). Coronal sections (14 μm) were cut using a freezing stage microtome and mounted on glass slides (Superfrost plus, Fisher). Seventy-two sections spanning the whole rostro-caudal extension of the PFC were collected for ISH. During the slice process, every other slice was collected in a 24-well plate, so each section was 28 μm from the slice collected before it. Sections were stored in PBS containing DEPC until processed for RNAscope/*In situ* hybridization (ISH; see below).

#### Probe hybridization

Probes and amplification/signal detection reagents were provided by (Advanced Cell Diagnostics, Newark, CA, USA). Hybridization was performed using a single probe assay kit (RNAscope 2.5 High-Definition BROWN Assay kit, Cat. # 322371) following the manufacturer’s user manual with minor modifications. Briefly, sections were treated with 0.9% H_2_O_2_ and 10% methanol in phosphate buffer saline for 10 min in 10 nM sodium citrate buffer solution (pH 6.0) for 2 min at 91°C. Sections were next treated with proteinase K (1 μg/ml) for 15 min at 40°C and then incubated using the Rn-Chrm1 probe (Advanced Cell Diagnostics, Cat. # 485301) for 2 h at 40°C. Signals were amplified using the single probe assay kit reagents. Before the first amplification step, sections were washed in 0.1× SSC buffer at 33°C. Sections were also washed in the same solution after each of the following six amplification steps. The first three times at 35°C, and then at room temperature. Signals were detected using DAB. Signal development was monitored under a microscope and stopped when the desired signal intensity was achieved. Sections were immediately further processed for immunostaining.

#### Immunostaining

ISH-labeled sections adhered to glass slides were washed in TBS for 10 min, repeated three times, treated for 1 h in TBS containing 3% NGS, 1% BSA, 0.2% Triton-X 100 and then incubated overnight with primary antibodies [Chicken-GFP, Abcam, Cambridge, United Kingdom, 1:1,000 diluted in TBS containing 1% normal goat serum, 1% bovine serum albumin (BSA), 0.2% Triton-X 100 at 4*^o^*C]. Staining was visualized by the ABC method using SG peroxidase substrate (Vector laboratories, Burlingame, CA, USA).

#### Image acquisition and quantification

For unbiased counting in the different cortical areas, bright field images were acquired using a Nikon DS-Fi3 digital camera connected to a (Nikon Instruments, Melville, NY, USA) Ti2 Widefield microscope. All images were processed with Adobe Photoshop CC or Adobe Illustrator CC. Brightness and contrast were adjusted to optimize automatic detection. M1-acetylcholine receptor expression was quantified in layer five of the PLC and the primary motor cortex (M1) by counting the number of DAB-stained mRNA puncta in each. Quantification was performed on four coronal sections from each animal. Puncta were quantified with an automated detection algorithm using Nikon NS-elements Advanced Research version. For quantification of mRNA expression in identified PLC commissural neurons, monochrome images were acquired with an Optronic camera mounted on a Zeiss upright microscope and using a 63X oil immersion objective lens. mRNA puncta in each retrogradely stained neuron were manually counted using Stereo Investigator (MBF Bioscience, Williston, VT, USA).

### Behavioral assessments

#### Cannulation

Rats were anesthetized with isoflurane (4%) and secured in a stereotaxic frame (David Kopf Instruments). Isoflurane concentration was reduced to 2% with a flow rate of 0.8 l/min. A stainless steel guide cannula (26 gauge) with a dummy probe was placed and fixed to the skull using Lang dental cement. A single cannula was positioned medially using the following stereotaxic coordinates AD: 2.5 mm, ML: 0.0 mm, and DV: 3.0 mm. Cannulated animals were used for behavioral tests 3 days after cannulation. Pirenzepine (2.5 nmoles) or saline control solution was infused (1 μl/min) 30 min prior to behavioral assessments.

#### Tactile allodynia (Von Frey test)

Seven days after SNI/sham surgery, tactile sensitivity of the hind paws was measured using Von Frey filaments. Animals were placed in a Plexiglass box with a perforated grid floor and given 30 min to habituate to the environment. Electronic Von Frey filament designed for rat paws application (Harvard Instruments, Holliston, MA, USA) electronically measured the force (in grams) applied to the lateral part of the plantar surface of the hind paw. The filament was applied for 2 s for three trials per hind paw, and paw withdrawal during the application was considered a positive response. Paw withdrawals due to locomotion or weight shifting were not counted and such trials were repeated. The 50% tactile threshold was calculated based on the average of three trials.

#### Cold sensitivity (acetone test)

Cold sensitivity was measured using the acetone drop test. A blunt needle connected to a syringe was used to apply one drop (∼10 μl) of acetone solution on the lateral plantar surface of the hind paw. The duration of paw withdrawal was recorded, and a score was given to categorize the response behavior (0 = no response, 1 = startled behavior, 2 = withdrawal of hind paw for <5 s, 3 = withdrawal of hind paw for >5 s, 4 = withdrawal for >30 s). Paw withdrawal due to locomotion or weight shifting was not counted. Trials were repeated three times per hind paw.

### Data representation and statistical analysis

The sample size of every experimental group is provided in the figure legends. Statistical calculations were performed using Prism 9 software (GraphPad Software, La Jolla, CA, USA). Whisker plots show median (lines inside boxes), 25th and 75th percentile (box margins), 10th–90th percentile (whiskers), and individual values (symbols). Bar plots show mean and SEM. Statistical significance was assessed using either unpaired or paired *t*-tests, Mann-Whitney test or two-way ANOVA, according to experimental design and sample properties (see figure legends).

## Results

### Excitatory cholinergic modulation of commissural L5 PLC neurons in male and female rats

**FIGURE 1:**
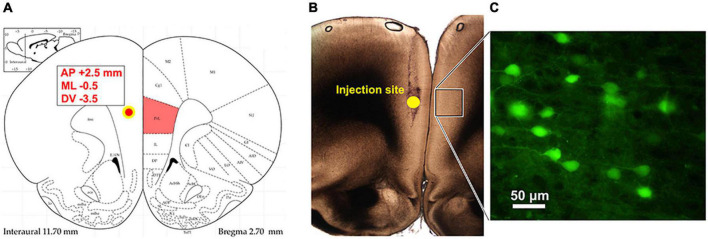
Identification of commissural PLC neurons using retrograde viral tracing. **(A)** Injection site of retrograde pAAV-hsyn-EGFP into the PLC ipsilateral to the peripheral injury. Stereotaxic coordinates to target the left prelimbic cortex were AP: +2.5 mm from bregma; ML: –0.5; DV: 3.5 mm ventral to the surface. **(B)** Photograph of the injection site and the tract left from Hamilton syringe. The box represents the contralateral cortex where GFP expression was detected. **(C)** Retrograde AAV-mediated EGFP expression in PLC neurons in the hemisphere contralateral to virus injection.

**FIGURE 2:**
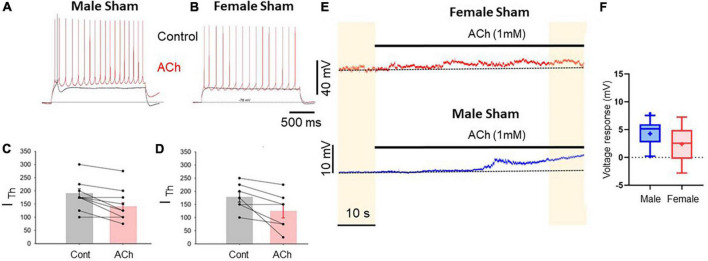
Prelimbic cortex L5 excitatory cholinergic modulation is similar in pyramidal cells of male and female rats. **(A,B)** Voltage traces recorded from L5 pyramidal neurons in acute PLC slices in response to depolarizing current injection in control conditions (black traces) and after 1 mM ACh (red traces) in slices from sham male **(A)** and female **(B)** rats. The bathing solution contained blockers of fast synaptic transmission (50 μM picrotoxin and 2.5 mM kynurenic acid). Resting potential was ∼–75 mV. **(C,D)** Summary plots showing the reduction in rheobase current caused by acetylcholine in male **(C)** and female **(D)** animals; no sexual dimorphism was detected in this response; *p* = 0.67. **(E)** Depolarizing responses caused by ACh application (horizontal bar) observed for 1 min in continuous recordings in slice from a female (top trace) and a male (bottom trace). **(F)** Summary plot representing the average ACh-dependent depolarization calculated comparing the voltage recorded in the first 10 s and the final 10 s of recording (yellow shaded areas). The mean ACh-mediated depolarization was 2.4 ± 1.59 mV in cells from females and 4.3 ± 0.72 mV in cells from males (males vs. females *p* = 0.22, Mann–Whitney test, 11 male and 5 female cells, respectively).

We previously showed that M1-mediated cholinergic excitation is impaired in layer five presumptive commissural (as established from *post hoc* firing patter analysis) pyramidal neurons of the medial prefrontal cortex of male SNI rats 1 week after peripheral neuropathic injury (Radzicki et al., 2017). Because an increasing wealth of data show large sexual dimorphism of the brain both in physiological and pathological conditions, including rearrangement of the medial prefrontal cortex in neuropathic pain conditions (Shiers et al., 2018; Jones and Sheets, 2020), we tested whether commissural pyramidal neurons in layer 5 of the prelimbic cortex of female and male rats show similar responses to cholinergic stimulation. To this end, female and male rats were infused with a retrograde viral construct expressing EGFP under the synapsin promoter unilaterally in the left PLC. After 3 weeks the same animals received either SNI or sham surgery on the left hind paw. The animals were then sacrificed after another week for electrophysiological interrogation of commissural layer five neurons, which were identified by expression of eGFP in the right cortex (Figure 1). We found that in slices from control (sham operated) rats, the responses of commissural pyramidal cells to focal bath application of acetylcholine (1 mM) were similar but not identical in male and female animals. The most striking effect of cholinergic modulation was the reduction of the amount of depolarizing current necessary to evoke spiking (rheobase current), which was detected in both females and males. In control animals, acetylcholine caused the rheobase current to shift from 190 ± 18.3 pA to 140.6 ± 14.9 pA in males (10 cells; *p* = 0.02) and from 178.6 ± 19.2 pA to 125 ± 26.2 pA in females (8 cells; *p* = 0.027; Figures 2A–D). In addition to this shift in the rheobase current, acetylcholine also caused a slow depolarization in the absence of any current stimulation. This depolarization was detectable in slices from both females and males, but it was more consistent in males (4.3 ± 0.72 mV in males vs. 2.4 ± 1.59 mV in females, *n* = 11 and 5, respectively, *p* = 0.22 Mann–Whitney test, Figures 2E, F).

#### Excitatory cholinergic modulation of commissural PLC neurons is abolished in both male and female SNI rats

**FIGURE 3:**
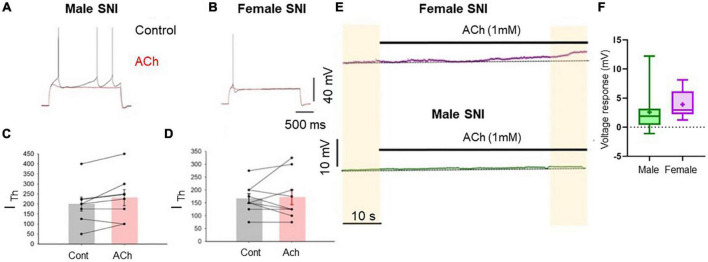
Cholinergic excitatory modulation of PLC pyramidal cells is abolished in SNI animals. Voltage traces recorded from L5 pyramidal neurons in acute slices from male **(A)** and female **(B)** SNI rats (1 week after peripheral injury) in the presence of blockers of fast synaptic transmission (50 μM picrotoxin and 2.5 mM kynurenic acid). Responses to 2-s-long injection of near-threshold depolarizing current in control conditions (black) and after 1 mM ACh (red). Resting potential was ∼–75 mV. ACh failed to increase cellular excitability. In males, ACh significantly decreased neuronal excitability, producing an increase in rheobase current from 200 ± 35.4 to 231.3 ± 40.3 pA **(C)**. In females, ACh did not significantly impact the input/output curve or the rheobase current **(D)**. **(E)** Voltage responses to ACh application in continuous recordings in slice from a female (top trace) and a male (bottom trace) rat at constant bias current injection. **(F)** Summary of the Ach-induced depolarization obtained in slices from SNI rats. The depolarization was 3.427 ± 0.25 mV in females and 0.554 ± 0.16 mV in males (*p* = 0.0037 for males vs. females, Mann-Whitney test, *n* = 8 and 9 cells, respectively).

In slices obtained from rats 7 days after SNI surgery an excitatory cholinergic response was detected neither in females nor in males. In slices from female rats, the rheobase current was not significantly affected by acetylcholine (Figures 3B, D). In contrast, recordings from slices from male SNI animals revealed that acetylcholine not only failed to excite layer five commissural neurons, but decreased their excitability, causing an increase in rheobase current (it was 200 ± 35.4 pA in control conditions and 231.3 ± 40.3 pA in the presence of acetylcholine, *n* = 8, *p* = 0.049, paired *t*-test; Figures 3A, C). Additionally, the slow depolarizing response was also abolished in male SNI animals (Figures 3E, F). These data show that excitatory cholinergic modulation of commissural layer five PLC differs between males and females, and that the SNI condition enhances the sexual dimorphism of the responses.

#### Neuropathic injury differentially regulates M1 expression in the PLC of male and female rats

**FIGURE 4:**
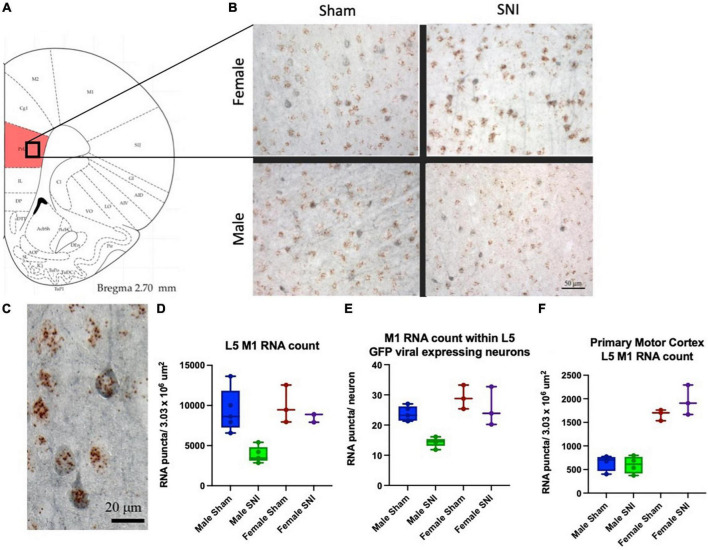
M1 expression in the PLC is differentially regulated in males and females. **(A)** Schematic representing the cortical area analyzed. **(B,C)** *In situ* hybridization for M1 RNA (brown dots) combined with immunohistochemistry targeting EGFP (blue) to identify PLC commissural neurons. One week after peripheral surgery, M1 RNA density in PLC L5 was reduced in male SNI animals compared to Sham (**D**; male SNI vs. sham, *p* = 0.003; female SNI vs. sham *p* = 0.36). When the quantification was limited to L5 commissural projecting neurons (eGFP positive neurons, blue), M1 RNA expression in SNI was also significantly reduced in male, but not female, rats [**(E)**, male SNI vs. sham, *p* ≤ 0.0001; female SNI vs. sham *p* = 0.46; two-way ANOVA]. **(F)** Control data were obtained in the same sections from the adjacent primary motor cortex and did not show any SNI-dependent change in M1 RNA expression (although there was a major sexual dimorphism in both control and SNI). Data from eight males (four Sham and four SNI) and six females (three Sham and three SNI); data points overlap in some plots.

We previously showed that the ACh-mediated depolarizing response in L5 PLC pyramidal neurons is mediated by M1 cholinergic receptors (Radzicki et al., 2017). Therefore, here we examined whether any difference exists in PLC M1 transcript expression between males and females in control and SNI rats. We performed *in situ* hybridization analysis of eight male and six female rats. To further quantify M1 expression selectively in commissural neurons, these studies were performed in virus-injected rats in which commissural neurons are identified by EGFP expression (Figures 4B, C). While no significant differences were observed between male and female animals in control (sham-operated) conditions, in SNI animals M1 expression was significantly reduced in male, but not in female, rats 1 week after the peripheral injury. When counting all layer five, M1 receptor expression in SNI PLC was reduced by 58.7% in males (Figure 4D), while in females the decrease was not significant (14.3% decrease, *p* = 0.36; Figure 4D). Similar dimorphism was observed when the count was restricted to commissural neurons only (EGFP-positive cells); in male SNI rats M1 expression in PLC commissural neurons was decreased by 39.7%, while in females no significant reduction could be detected (12.1% decrease; Figure 4E). Thus, at 7 days post-neuropathic injury, M1 subunit expression is decreased in the PLC of male but not female rats. In order to establish the network selectivity of these findings, we also quantified M1 subunit expression in the primary motor cortex, which is not part of the limbic system. The primary motor cortex was used as control also because it is contained within the same sections as the PFC and therefore allows a direct comparison in identical experimental conditions. As expected, no difference in M1-receptor expression was detectable between SNI and sham-operated animals in primary motor cortex, although the expression differed greatly between male and female rats in both control and SNI conditions (Figure 4F).

#### Selective blockade of PLC M1 receptors causes cold sensitivity in rats of both sexes

**FIGURE 5:**
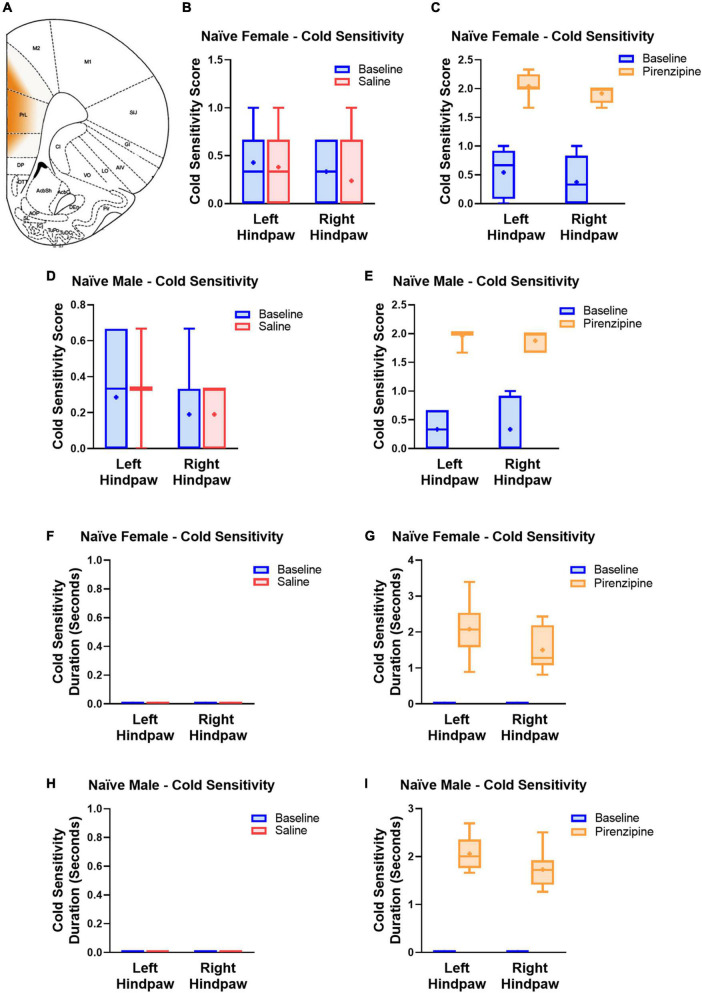
Selective pharmacological M1 blockade induces cold sensitivity in both males and females. **(A)** Schematic representing the cortical area where the drug was infused. Responses to the acetone test were quantified using a response score (top, **B–E)** and response duration (bottom, **F–I)**. Animals were first tested at baseline (blue bars) and 30 min later infused with 1 μl of either saline (7 male and 7 female control animals, red bars, **B,D,F,H**) or pirenzepine (2.5 nanomoles; experimental group, 8 male and 8 female rats, yellow bars, **C,E,G,I**) and re-tested. Note the pro-algesic effect of pirenzepine infusion in both males (8 rats) and females (8 rats). Statistical analysis using paired *t*-tests yielded the following *p*-values: Female Score Saline: *LP* = 0.6036; *RP* = 0.3559 (*n* = 7); Female Score pirenzepine: *LP* = 0.0001; *RP* = < 0.0001 (*n* = 8); Male Score Saline: *LP* = 0.6036; *RP* = > 0.9999 (*n* = 7); Male Score pirenzepine: *LP* = < 0.0001; *RP* = 0.0001 (*n* = 8); Female Duration Saline: n/a; Female Duration pirenzepine: *LP* = < 0.0001; *RP* = 0.0002; Male Duration Saline: n/a; Male Duration pirenzepine: *LP* = < 0.0001; *RP* = < 0.0001. RP and LP indicate tests on the right and left hind paw, respectively. Data points are aligned in a single file fashion. Consequently, dots with similar value are overlapping.

**FIGURE 6:**
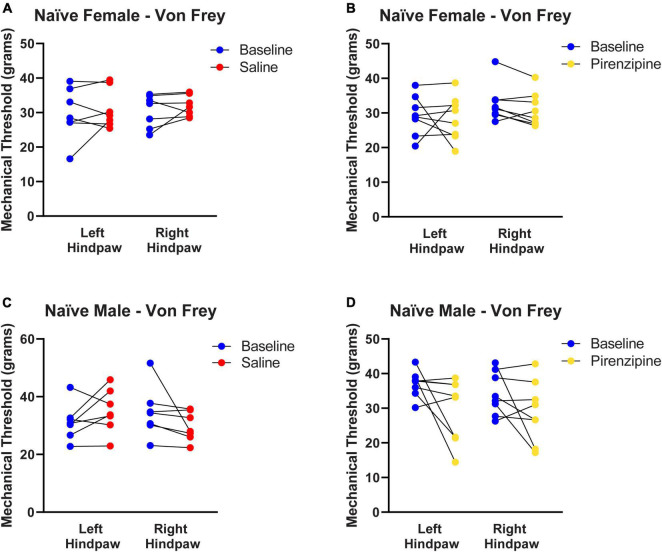
Selective pharmacological M1 blockade does not induce mechanical allodynia. Control **(A,C)** and experimental **(B,D)** animals were each tested for baseline thresholds (blue symbols). They were then infused with 1 μl of either saline (red symbols) or pirenzepine (yellow symbols) and re-tested 30 min after infusion. Pirenzepine did not have any significant on mechanical allodynia. Statistical analysis using paired *t*-tests yielded the following *p*-values: Female Saline: *LP* = 0.5140, *RP* = 0.3214 (*n* = 7). Female pirenzepine: *LP* = 0.7905, *RP* = 0.1114 (*n* = 8). Male Saline: *LP* = 0.1990, *RP* = 0.1666 (*n* = 7). Male pirenzepine: *LP* = 0.0901, *RP* = 0.1799 (*n* = 8). These are the same animal cohorts as in Figure 5. Data points are aligned in a single file fashion. Consequently, dots with similar value are overlapping.

We hypothesized that the impaired cholinergic excitability of PLC pyramidal neurons contributes to the global PLC deactivation in neuropathic pain (Jefferson et al., 2021). If that is the case, selective pharmacological M1 antagonism may also have pro-algesic effects in naïve animals. To this end 30 animals [14 control (saline injected) and 16 experimental animals (pirenzepine injected)] were cannulated in the PLC (Figure 5A) to allow focal mPFC delivery of the M1 antagonist pirenzepine (2.5 nmoles). We then tested the presence of cold sensitivity 30 min after pirenzepine infusion. We measured cold sensitivity score (see Materials and methods; Figures 5B–E) and duration (Figures 5F–I) and found that rats developed cold sensitivity, which was never detected in saline injected rats. The cold sensitivity was present in both hind paws in females (Left: from 0.54 ± 0.14 to 2.04 ± 0.08; right: from 0.38 ± 0.15 to 1.91 ± 0.05; *n* = 8; Figure 5C) and males (Figure 5E). Interestingly, cold selectivity scores produced by PLC M1 antagonism are only slightly lower than those in SNI mice (see Supplementary Figure 1). Thus, the PLC inhibition obtained by M1 antagonism is sufficient to cause cold sensitivity. Finally, we tested the effect of PLC infusion with pirenzepine on mechanical sensitivity but did not detect any significant effect in either female (Figures 6A, B) or male rats (Figures 6C, D).

## Discussion

Our study produced several novel findings. First, we found that excitatory cholinergic modulation of layer five commissural neurons of the PLC is similar but not overlapping in female and male rats. Second, we determined that in PLC slices from SNI rats, cholinergic excitability is more severely impaired in males than in females. Third, we discovered that the SNI-associated alteration of M1 subunit expression in the PLC is stronger in males than in females. Finally, we showed that in naïve rats selective pharmacological blockade of PLC M1 receptors is sufficient to cause cold allodynia in both males and females.

### Sex-specific differences in PLC cholinergic modulation in SNI animals

Cholinergic modulation increases mPFC excitability *in vivo* (Gill et al., 2000) and *ex vivo* (Radzicki et al., 2017). At the same time, deactivation of the mPFC constitutes a critical mechanism driving different symptoms of neuropathic pain (Ji et al., 2010; Zhang et al., 2015). Multiple convergent mechanisms are believed to contribute to the mPFC deactivation in pain, including altered synaptic inputs, increased activity of parvalbumin-positive GABAergic interneurons, and decreased intrinsic excitability (Wang et al., 2015; Zhang et al., 2015; Kelly et al., 2016; Cheriyan and Sheets, 2020). Interestingly, muscarinic modulation of cortical activity shows sexual dimorphism. For example, prelimbic cortex infusion with scopolamine, a wide spectrum muscarinic antagonist, impaires cued and contextual fear memories in male, but not female, rats (Kirry et al., 2019). Our data show that 1 week after peripheral injury muscarinic excitation is more severely impaired in male compared to female rats, although selective M1 blockade in the mPFC is sufficient to cause cold allodynia in naïve rats of both sexes. The stronger impairment of cholinergic PLC excitability in male neuropathic pain rats is in line with the observation that in neuropathic pain cognitive flexibility impairment is deeper in male than in female mice (Shiers et al., 2018) and in keeping with the stronger decrease in M1 transcript expression in male SNI (Figures 4D, E). The increased susceptibility of the male PLC to neuropathic pain-induced alterations is also reminiscent of a recent finding showing that excitability of PLC layer five parvalbumin-positive interneurons is increased in male, but not female, mice (Jones and Sheets, 2020). One point that remains unclear, however, is the reason for the larger depolarizing response to acetylcholine in cells from male sham compared to female sham animals (Figure 2F) despite no difference in M1 RNA expression (Figures 4D, E). One possible explanation is that M1 receptors can be coupled with different effectors (ion channels) in L5 mPFC neurons, as both TRPM and Nav1.9 channels contribute to the depolarizing response (Lei et al., 2014; Kurowski et al., 2015). The difference in the response to ACh between male and female neurons could be due to a differential expression of ion channels coupled with M1. Alternatively, the coupling pathways between receptor and effector may differ. More work will be required to verify this hypothesis.

### Potential mechanism of decreased M1 AChR expression in the PLC of SNI rats

A question that remains unanswered concerns the cellular mechanisms leading to the reduced M1 expression in the PLC. One possibility is that cholinergic inputs from the basal forebrain are increased, and that M1 downregulation represents receptor desensitization. This is in line with previously reported increased receptor internalization in the mPFC of SNI rats (Radzicki et al., 2017). It is, however important to keep in mind that the altered cholinergic response is far from the only change in layer five mPFC pyramidal neurons, as they show important physiological and morphological changes in neuropathic pain (Kelly et al., 2016; Shiers et al., 2018) and the whole mPFC gene expression patterns are profoundly altered in SNI animals (Alvarado et al., 2013). Because many of these gene expression changes are associated with alterations in DNA methylation profile (Tajerian et al., 2013) it is possible that the decreased M1 expression is independent of alterations in specific inputs and the consequence of general reduction in neuronal excitability, which has been shown to influence methylation (Sharma et al., 2008). Additionally, it is important to note that many of the mPFC alterations associated with neuropathic pain are cell-type and circuit specific; for example excitability of neurons projecting to the periaqueductal gray is reduced in prelimbic (PL), but not infralimbic (IL) cortex (Cheriyan and Sheets, 2018). Therefore, future studies will have to address whether the alterations in cholinergic modulation are limited to PLC or extend to other mPFC areas and to the different populations of interneurons.

### Differential regulation of cold and mechanical allodynia by M1 antagonism

Our finding that pharmacological M1 blockade in the mPFC is sufficient to induce cold allodynia in naïve rats suggests that the known analgesic effects of cholinergic agonists (Naser and Kuner, 2018) do not depend exclusively on spinal cord effects (Eisenach, 1999), but are at least in part the result of cortical modulation. The observation that mPFC infusion of pirenzepine causes thermal, but not mechanical, allodynia is also interesting. First, it shows that even a mild modulation of the mPFC has major impact on pain perception. It also supports a high basal activation of M1 receptors when the animal is not engaged in any specific cognitive task.

The divergence of mechanical and cold allodynia is not new, as these show different sexual dimorphism and are differentially modulated by treatment. For example, 7 days after SNI surgery, cold allodynia was detected in both male and female Sprague–Dawley rats, while mechanical allodynia at this time point was only significant in females (Ahlstrom et al., 2021). Various treatments may also differentially affect these two types of allodynia. Intrathecal injection of adipose tissue-derived stem cells in rats selectively reduces cold, but not mechanical, allodynia following L5 spinal nerve ligation (Jwa et al., 2020). On the contrary, electrical stimulation of the spinal cord of SNI rats was found to reduce injury-induced mechanical allodynia without affecting cold allodynia (Tao et al., 2021). Additional differences exist between rodent species. For example, while most rodents develop robust mechanical and cold allodynia after SNI surgery, naked mole-rats do not develop cold allodynia (Poulson et al., 2020). While the cellular mechanisms potentially involved in this divergence remain unknown, it is possible to suggest a modulation of the inputs from the central amygdala because previous work showed that pharmacological inactivation of this structure using lidocaine under some circumstances ameliorated mechanical but not cold allodynia (Cooper et al., 2018). Thus, numerous different mechanisms likely account for this differential modulation. Finally, it is worth noting that all our behavioral assessments were performed during the light phase of the day. Because acetylcholine release is stronger during wakefulness than during deep sleep (Saper and Fuller, 2017), and the observed effects were the result of M1 antagonism, it is possible that they represent a lower limit of the response range, and that effects are stronger if animals are tested during the dark (wakefulness) cycle.

## Data availability statement

The raw data supporting the conclusions of this article will be made available by the authors, without undue reservation.

## Ethics statement

The animal study was reviewed and approved by the Northwestern University Institutional Animal Care and Use Committee.

## Author contributions

TJ performed the experiments, analyzed the data, prepared the figures, and contributed to writing the manuscript. HK analyzed data (including MATLAB code writing), contributed to making the figures, and provided critical reading of the manuscript. MM designed the project and the experiments, contributed to data analysis, and drafted the manuscript. All authors contributed to the article and approved the submitted version.
