# Supplementary figures and images for: Impaired muscarinic modulation of the rat prelimbic cortex in neuropathic pain is sexually dimorphic and associated with cold allodynia
Source: Front Cell Neurosci. 2023 Feb 9;17:984287. doi: 10.3389/fncel.2023.984287 (PMC9947152; doi:10.3389/fncel.2023.984287)

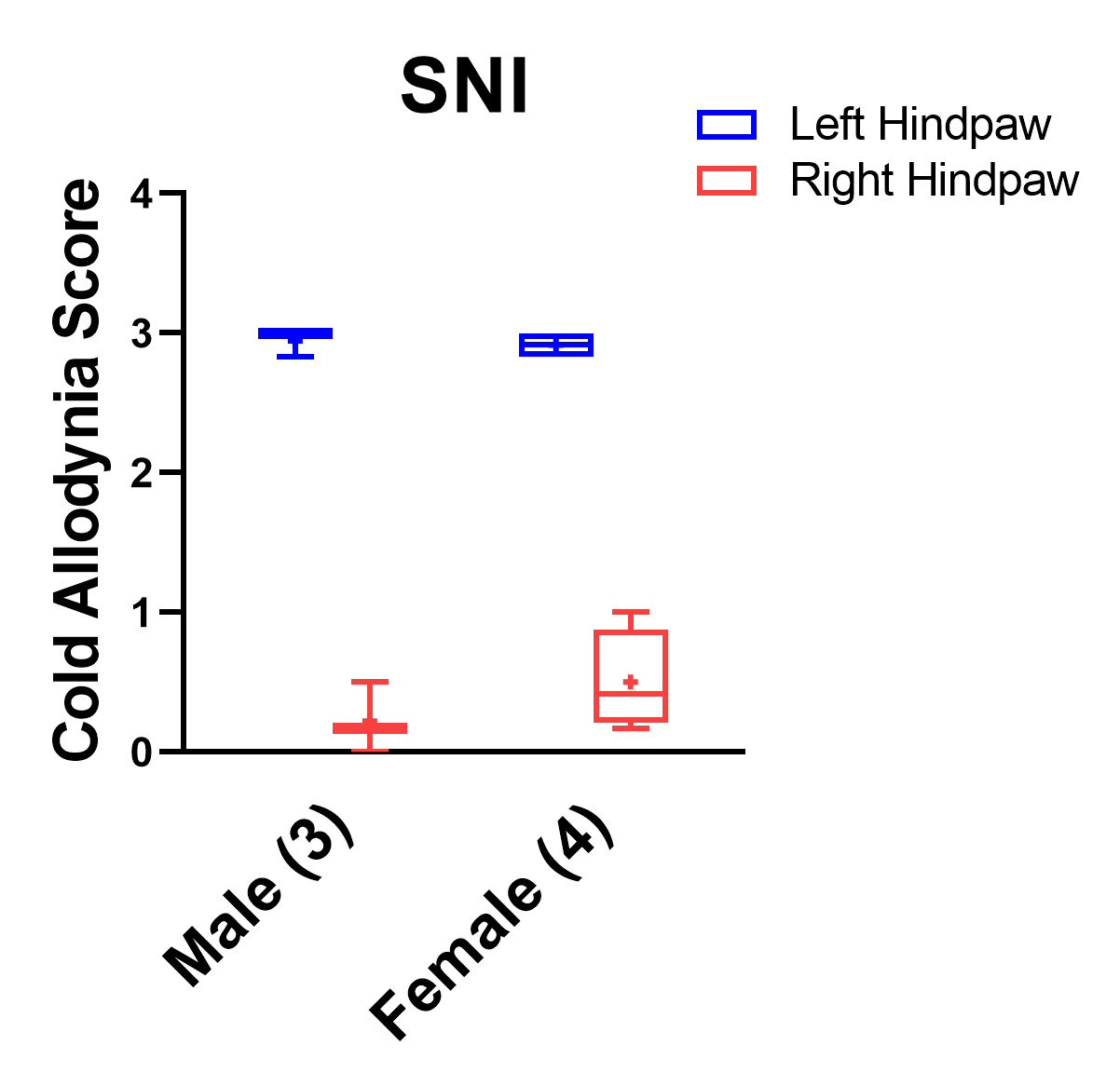

Supplement: Supplementary file 1 [file Image_1.TIF]
