# Supplementary material for: Impaired muscarinic modulation of the rat prelimbic cortex in neuropathic pain is sexually dimorphic and associated with cold allodynia
Source: Front Cell Neurosci. 2023 Feb 9;17:984287. doi: 10.3389/fncel.2023.984287 (PMC9947152; doi:10.3389/fncel.2023.984287)
Supplement: Supplementary file 2 [file Table_1.DOCX]

Supplementary Material

# Supplementary tables

1. **Reagents and critical materials**

| REAGENT or RESOURCE | SOURCE | IDENTIFIER |
| --- | --- | --- |
| **Antibodies** | | |
| Chicken polyclonal GFP antibody (1:1000) | Abcam | Cat.# ab13970, RRID:AB_300798 |
| Rabbit GFP antibody (1:500) | Millipore | Cat.# AB3080, RRID:AB_91337 |
| **Bacterial and Virus Strains** | | |
| pAAV-hSyn-EGFP | Addgene | RRID:Addgene_50465 |
| **Chemicals, Peptides, and Recombinant Proteins** |  |  |
| Proteinase K | Sigma-Aldrich | Ref. 0311588701 |
| SG substrate kit peroxidase | Vector laboratories | Cat.# SK-4700, RRID:AB_2314425 |
| Vectamount | Vector laboratories | Cat.# H-5000, RRID:AB_2336786 |
| **Critical Commercial Assays** | | |
| RNAscope 2.5 High Definition BROWN Assay kit | Advanced Cell Diagnostics, USA | Cat.# 322371 |
| **Oligonucleotides** |  |  |
| Rn-Chrm1 | Advanced Cell Diagnostics, USA | Cat. # 485301 |
| **Software and Algorithms** |  |  |
| Clampfit9.0 | Axon Instruments | www.moleculardevices.com |
| NIS-Elements | Nikon Instruments Inc. | www.microscope.healthcare.nikon.com |
| Stereo Investigator | MBF Bioscience | www.mbfbioscience.com |
| MATLAB | MathWorks | www.mathworks.com |
| Prism9 | GraphPad | www.graphpad.com |
| Other | | |
| Temperature transponder | Warner Instrument Co., USA | TC324B |

1. **Statistical assessments**

| **Experiment** | **Comparison between…** | **Statistical test** | **Sample size** | **p value** |  |  |
| --- | --- | --- | --- | --- | --- | --- |
| Steady state voltage response following onset of ACh | SHAM Male vs Female | Mann-Whitney | Males = 11; Females = 5 | 0.2211 |  |  |
| Steady state voltage response following onset of ACh | SNI Male vs Female | Mann-Whitney | Males = 9; Females = 9 | 0.4042 |  |  |
| RNAscope M1 L5 of PLC | SHAM vs SNI (Male) | unpaired t test | Sham = 4; SNI = 4 | 0.0027 |  |  |
| RNAscope M1 L5 of PLC | SHAM vs SNI (Female) | unpaired t test | Sham = 3; SNI = 3 | 0.3618 |  |  |
| RNAscope M1 Commissural L5 neurons | SHAM vs SNI (Male) | unpaired t test | Sham = 4; SNI = 4 | <0.0001 |  |  |
| RNAscope M1 Commissural L5 neurons | SHAM vs SNI (Female) | unpaired t test | Sham = 3; SNI = 3 | 0.4629 |  |  |
| RNAscope M1 L5 Primary Motor Cortex | SHAM vs SNI and Male vs Female | Two-Way ANOVA | Males: Sham = 4; SNI = 4/ Females: Sham = 3;SNI = 3 | Sex = <0.0001; Condition = 0.4141 |  |  |
| RNAscope M1 L5 Intralimbic Cortex | SHAM vs SNI and Male vs Female | Two-Way ANOVA | Males: Sham = 4; SNI = 4/ Females: Sham = 3;SNI = 3 | Sex = 0.0002; Condition = 0.0224 |  |  |
| RNAscope M1 L5 Anterior Cingulate Cortex | SHAM vs SNI and Male vs Female | Two-Way ANOVA | Males: Sham = 4; SNI = 4/ Females: Sham = 3;SNI = 3 | Sex = 0.0014; Condition = 0.0046 |  |  |
| Naïve Female Von Frey (Saline) | Baseline vs Treated | paired t test | n = 7 | Left Hindpaw (LP) = 0.5140; Right Hindpaw (RP) = 0.3214 | | |
| Naïve Male Von Frey (Saline) | Baseline vs Treated | paired t test | n = 7 | LP = 0.1990 ; RP = 0.1666 |  |  |
| Naïve Female Von Frey (2.5 nm Pirenzipine) | Baseline vs Treated | paired t test | n = 8 | LP = 0.7905 ; RP = 0.1114 |  |  |
| Naïve Male Von Frey (2.5 nm Pirenzipine | Baseline vs Treated | paired t test | n = 8 | LP = 0.0901; RP = 0.1799 |  |  |
| Naïve Female Cold Allodynia **Score** (Saline) | Baseline vs Treated | paired t test | n = 7 | LP = 0.6036; RP = 0.3559 |  |  |
| Naïve Female Cold Allodynia **Score** (Pirenzipine) | Baseline vs Treated | paired t test | n = 8 | LP = 0.0001; RP = <0.0001 |  |  |
| Naïve Female Cold Allodynia **Duration** (Saline) | Baseline vs Treated | paired t test | n = 7 | N/A |  |  |
| Naïve Female Cold Allodynia **Duration** (Pirenzipine) | Baseline vs Treated | paired t test | n = 7 | LP = <0.0001; RP = 0.0002 |  |  |
| Naïve Male Cold Allodynia **Score** (Saline) | Baseline vs Treated | paired t test | n = 7 | LP = 0.6036; RP = >0.9999 |  |  |
| Naïve Male Cold Allodynia **Score** (Pirenzipine) | Baseline vs Treated | paired t test | n = 8 | LP = <0.0001; RP = 0.0001 |  |  |
| Naïve Male Cold Allodynia **Duration** (Saline) | Baseline vs Treated | paired t test | n = 7 | N/A |  |  |
| Naïve Male Cold Allodynia **Duration** (Pirenzipine) | Baseline vs Treated | paired t test | n = 8 | LP = <0.0001; RP = <0.0001 |  |  |
